# Gα13 Promotes Clonogenic Growth by Increasing Tolerance to Oxidative Metabolic Stress in Prostate Cancer Cells

**DOI:** 10.3390/ijms26104883

**Published:** 2025-05-20

**Authors:** Di Wu, Wei Kiang Lim, Xiaoran Chai, Veerabrahma Pratap Seshachalam, Suhail Ahmed Kabeer Rasheed, Sujoy Ghosh, Patrick J. Casey

**Affiliations:** 1 Program in Cancer and Stem Cell Biology, Duke-NUS Medical School, 8 College Road, Singapore 169857, Singapore; 2 Program in Cardiovascular and Metabolic Disorders, Duke-NUS Medical School, 8 College Road, Singapore 169857, Singapore; 3 Program in Clinical and Translational Liver Cancer Research, Division of Medical Science, National Cancer Center Singapore, 30 Hospital Boulevard, Singapore 168583, Singapore; 4 Laboratory of Functional Genomics, Pennington Biomedical Research Center, 6400 Perkins Road, Baton Rouge, LA 70808, USA; 5 Department of Pharmacology and Cancer Biology, Duke University Medical Center, 308 Research Drive, Durham, NC 27710, USA

**Keywords:** Gα13, *GNA13*, prostate cancer, superoxide, superoxide dismutase, SOD2, oxidative metabolic stress, mitochondria, LNCaP, PC3

## Abstract

The oncogenic role of the G12 family in many human solid cancers has been extensively studied, primarily through the effects of constitutively active mutants of these proteins on cell migration and invasion. However, these mutations are not seen in cancers, and the biological role of Gα13 in prostate cancer tumorigenesis is largely unexplored. Here, we report that Gα13 promotes anchorage-independent colony formation, spheroid formation, and xenograft tumor growth in human prostate cancer cell lines. Transcriptome analyses suggest that Gα13 modulates genes in the mitochondria and are involved in the oxidative stress response. Silencing of *GNA13* increased mitochondrial superoxide levels when prostate cancer cells were cultured in galactose medium and increased the sensitivity to oxidative metabolic stress when the cells were cultured in media containing non-glycolytic metabolites. Furthermore, Gα13 levels impacts the abundance of superoxide dismutase 2 (SOD2) in the mitochondria, as well as *SOD2* promoter activity and mRNA expression. Importantly, expression of SOD2 could rescue the effect of Gα13 loss on suppression of anchorage-independent growth. Likewise, stable knockdown of *SOD2* decreased anchorage-independent cell growth, which was enhanced by overexpression of Gα13. These results outline a novel biological function of Gα13 mediated via SOD2 in prostate cancer tumorigenesis and highlight it as a potential treatment target.

## 1. Introduction

Gα13 and Gα12 are members of the G12 family of Gα proteins that, along with their associated Gβγ subunits, mediate signaling from specific G protein-coupled receptors (GPCRs). The oncogenic role of Gα13 in several human cancers has been extensively studied and can be distinct from that of Gα12 [1,2,3]. Analysis of patient samples indicate that Gα13 expression correlates with survival in solid cancers such as gastric [4], hepatocellular [5], esophageal [6] and head and neck [7] cancers. In vitro studies showed a role for Gα13 in cell migration, invasion, drug resistance, and tumor-initiating phenotypes in human solid cancer cell lines [5,7,8,9,10,11,12,13,14,15,16,17,18,19]. Our laboratory recently published a review on the role of GPCR–Gα13 in mitochondrial function, oxidative stress, and prostate cancer [20] and identified a gap in knowledge: the role of Gα13 in prostate tumorigenesis and on other cellular processes such as energy metabolism and cell survival has not been explored [8,15]. Furthermore, while there are no activating mutations of *GNA13* in androgen-independent metastatic prostate cancer, gene amplification of wild-type *GNA13* is seen in 4.4% (19/429) of cases [21].

An unusual role of Gα13 is on in the oxidative stress response, where several studies have found an effect of GPCR–Gα13 signaling on the activation of transcription factor nuclear factor erythroid 2-related factor 2 (Nrf2) and antioxidant gene promoters in mouse fibroblasts [22,23]. Recently, NRF2 has been shown to act on the promoter of the superoxide dismutase 2 (SOD2) gene by ChIP-qPCR analysis and to increase its mRNA and protein expression in human lung cancer cells [24,25]. Interestingly, patient data support the notion that superoxide dismutase 2 (SOD2) may function in oncogenesis in some prostate cancers. SOD2 is a member of the SOD family of antioxidant enzymes that catalyze the transformation of superoxide to H_2_O_2_. There are three forms of SOD that are expressed in separate cellular compartments: cytoplasmic and nuclear SOD1, mitochondrial SOD2, and extracellular SOD3. The SOD2 protein is significantly increased in prostate tumors (Gleason 3–9) compared to hyperplastic or normal tissue [26], and its expression level correlates with prognostic Gleason scores [27]. Control samples and low-grade tumors (Gleason 5) have much lower SOD2 levels than medium grade tumors (Gleason 7), and high-grade tumors (Gleason 8) have even higher SOD2 protein levels [27]. Moreover, the SOD2 (rs4880) polymorphism increases prostate cancer risk [28,29,30] and the odds for high-grade tumors [31]. However, homozygous SOD2 (rs4880) was not predictive of prostate cancer recurrence [32] or overall survival [29,33] after radical prostatectomy. In vitro, increased SOD2 protein levels are observed upon neuroendocrine differentiation [27] and confer resistance to irradiation [34]. In this study, we revealed a novel biological function of Gα13 that functions in part through regulation of mitochondrial SOD2 in prostate cancer tumorigenesis.

## 2. Results

### 2.1. Gα13 Expression Increases Clonogenic Growth in PC3 and LNCaP Cells

**Figure 1:**
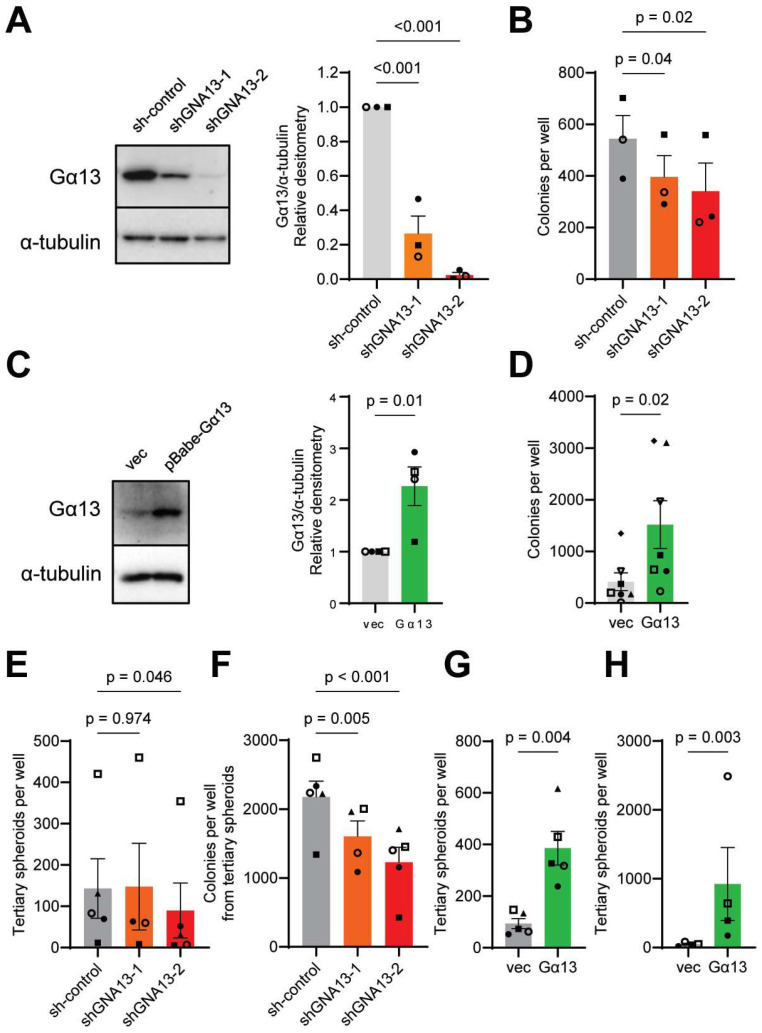
**Modulation of Gα13 expression impacts 3D anchorage-independent growth in PC3 and LNCaP cells.** (**A**,**C**) Gα13 protein levels in (**A**) PC3 and (**C**) LNCaP cells. (**B**,**D**) Number of colonies formed in 3D soft agar upon Gα13 modulation in (**B**) PC3 and (**D**) LNCaP cells. (**E**,**G**) Number of tertiary spheroids and (**F**,**H**) 3D colonies from tertiary spheroids upon Gα13 modulation in (**E**,**F**) PC3 and (**G**,**H**) LNCaP cells. Data points represent independent experiments. (**A**,**C**) *p* values calculated by unpaired, two-tailed *t*-tests or unpaired one-way ANOVA with Dunnett’s post hoc test. (**B**,**D**,**E**–**H**) *p* values calculated by paired, two-tailed *t*-tests or paired, one-way ANOVA with Dunnett’s post hoc test.

We previously reported that Gα13 protein levels correlate with cell migration and invasive phenotypes in prostate cancer cell lines [8,15]. Here, we used the same prostate cancer cell lines PC3, which exhibits relatively high levels of Gα13 protein expression, and LNCaP, which has lower levels of Gα13 protein, to further investigate the role of Gα13 in prostate cancer cell growth. In PC3 cells, two independent shRNAs against *GNA13* (sh*GNA13*-1 and sh*GNA13*-2) suppressed Gα13 protein levels by approximately 60% and 90%, respectively [Figure 1A], and reduced soft agar colony formation [Figure 1B], and this was partially rescued by re-expression of Gα13 [Supplementary Figure S1A]. Similarly, overexpression of Gα13 in LNCaP cells promoted soft agar colony growth [Figure 1C,D]. In contrast, modulating Gα13 expression in PC3 and LNCaP cells had little to no impact on short-term monolayer proliferation [Supplementary Figure S1B–D]. These results suggest that Gα13 contributes to tumorigenesis in the prostate cancer cell lines PC3 and LNCaP.

To further assess the role of Gα13 in clonogenic growth, we generated spheroids in 125 µg/mL of Matrigel [Supplementary Figure S1E] [35,36]. Upon serial replating, loss of Gα13 in PC3 cells reduced tertiary spheroid formation and suppressed colony formation from tertiary spheroids [Figure 1E,F]. Stable expression of sh*GNA13* maintained *GNA13* knockdown throughout the experiment [Supplementary Figure S1F]. Similarly, overexpression of Gα13 in LNCaP significantly increased tertiary spheroid formation and colony formation [Figure 1G,H]. These findings indicate that Gα13 contributes to processes involved in tumor initiation in the prostate cancer cell lines PC3 and LNCaP.

To further support our findings, exome sequencing datasets showed that the *GNA13* gene was amplified in 4% (21/492) of primary prostate adenocarcinoma samples in The Cancer Genome Atlas (TCGA) and in 28% (123/444) of metastatic castration-resistant prostate cancers (CRPCs) in the SU2C/PCF datasets [Supplementary Figure S2A] [20,21,37]. In addition, *GNA13* mRNA levels were positively and significantly correlated with prognostic Gleason scores in the TCGA-PRAD and SU2C/PCF datasets [Supplementary Figure S2B].

### 2.2. Gα13 Expression Modulates Mitochondrial SOD2 Expression

**Figure 2:**
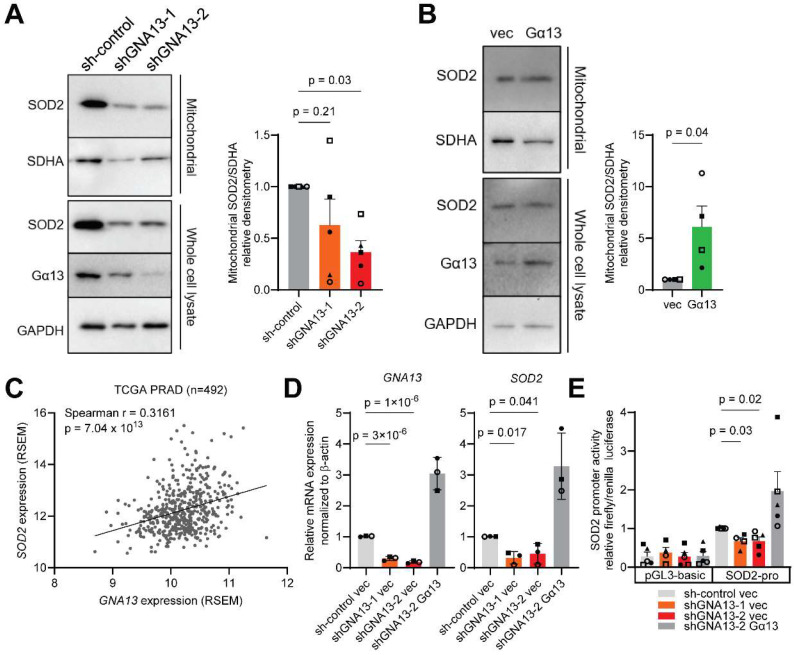
**The association between Gα13 and mitochondrial SOD2 expression.** (**A**,**B**) Effect of Gα13 on mitochondrial SOD2 protein levels in (**A**) PC3 and (**B**) LNCaP cells in representative Western blots. (**C**) Correlation plot of *GNA13* against SOD2 mRNA expression from TCGA database of prostate cancer patient (PRAD) tumor samples extracted from cBioPortal. Correlations were calculated by Spearman’s Rank Correlation for the correlation coefficient rho. (**D**) Effect of Gα13 on SOD2 mRNA expression in PC3 cells. (**E**) Effect of Gα13 on SOD2 promoter activity in PC3 cells. The ratio of Firefly to Renilla luciferase activity was calculated, and fold change relative to sh-control cells is shown. (**A**,**B**,**D**,**E**) Data points represent independent experiments. *p* values calculated by unpaired, two-tailed *t*-tests or unpaired one-way ANOVA with Dunnett’s post hoc test.

Explorative transcriptome analyses suggested that Gα13-regulated genes are enriched in the mitochondria. Amongst these genes, we prioritized the SOD2 gene as it has been reported to correlate with prostate cancer risk [28,29,30] and prognostic Gleason scores [26,27] [Supplementary Figure S3]. Furthermore, SOD2 has been shown to be an early response gene to anchorage-independence [38,39]. Hence, to augment the effect of Gα13-mediated SOD2 expression, we used ultra-low adherent (ULA) cell culture conditions to model short-term anchorage independence. Also, it has been reported that the Gα13 lower-expressing LNCaP cells have slightly lower SOD2 protein levels compared to the Gα13 higher-expressing PC3 cells [40,41]. To begin to assess the impact of Gα13 on mitochondrial SOD2, we first silenced *GNA13* in PC3 cells and then isolated the mitochondria by centrifugation. Silencing of *GNA13* in PC3 cells reduced mitochondrial SOD2 protein levels under ULA conditions [Figure 2A]. Likewise, overexpression of Gα13 increased SOD2 protein levels in LNCaP cells [Figure 2B]. We further verified that modulation of Gα13 expression in PC3 and LNCaP cells did not significantly change the mitochondrial mass, as assessed by mitochondrial outer membrane markers TOMM20 and VDAC and MitoView Green staining [Supplementary Figures S4 and S5]. Hence, it is unlikely that the impact of Gα13 on mitochondrial SOD2 protein levels is due to changes in the mitochondrial mass.

Then, to examine whether the impact of Gα13 on SOD2 protein expression might be mediated through SOD2 mRNA expression in prostate cancer tissue, we first analyzed three independent RNA sequencing datasets from TCGA-PRAD [42], the German Cancer Research Center (DKFZ, early-onset prostate cancer) [43] and the SU2C-PCF project (metastatic, castration-resistant prostate cancer) [44] on the cBioPortal website [37,45]. These analyses found strong and consistent positive correlations between *GNA13* and *SOD2* mRNA expression (Figure 2C, Supplementary Figure S6A,B). We further investigated the relationship between *GNA13* and *SOD2* in vitro. Stable knockdown or transient silencing of *GNA13* suppressed *SOD2* mRNA levels in PC3 cells [Figure 2D, Supplementary Figure S6C]. Rescue of Gα13 expression in PC3 sh*GNA13*-2 cells significantly increased *SOD2* mRNA levels [Figure 2D], but overexpression of Gα13 slightly but insignificantly increased *SOD2* mRNA levels in LNCaP cells [Supplementary Figure S6D]. We then constructed a luciferase reporter to evaluate the effect of Gα13 on *SOD2* promoter activity (-2500~+500 bp from the transcription start site). Stable knockdown of *GNA13* reduced *SOD2* promoter activity, which was rescued by Gα13 re-expression in PC3 cells [Figure 2E]; overexpression of Gα13 also slightly increased *SOD2* promoter activity in LNCaP cells [Supplementary Figure S6E]. Taken together, these data indicate that Gα13 regulates *SOD2* promoter activity and its mRNA and protein expression in the prostate cancer cell lines PC3 and LNCaP.

### 2.3. Gα13 Loss Increases Mitochondrial Superoxide Levels and Sensitivity to Oxidative Metabolic Stress in PC3 Cells

**Figure 3:**
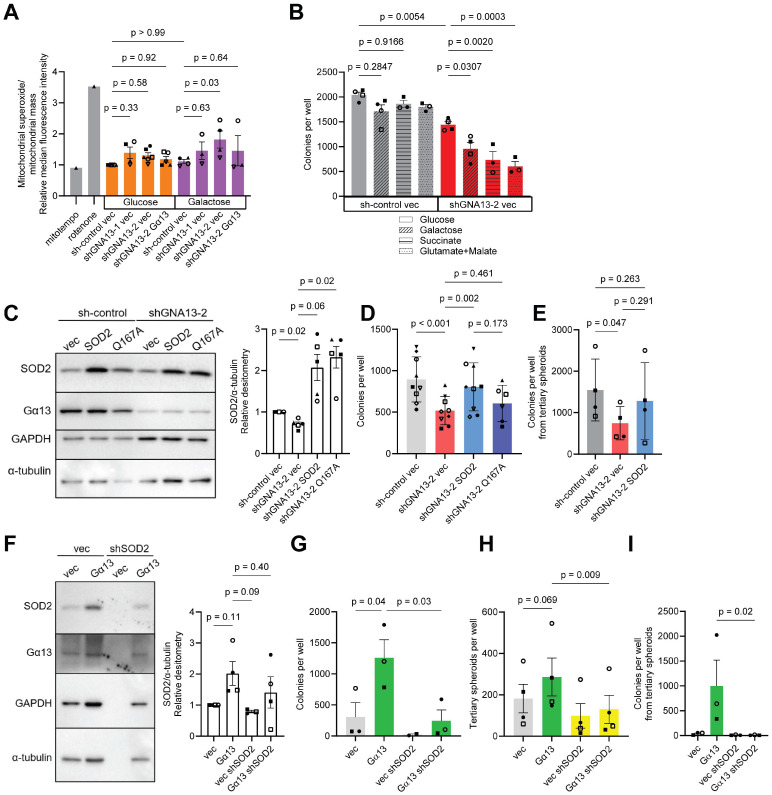
**Loss of Gα13 increases sensitivity to oxidative metabolic stress and decreases 3D colony formation.** (**A**) Effect of Gα13 on mitochondrial superoxide levels in PC3 cells in 11 mM glucose or 10 mM galactose media. Relative mitochondrial superoxide to mitochondrial mass ratio was calculated by median fluorescence intensity (MFI) of MitoSOX Red divided by MFI of MitoView Green. PC3 sh-control cells were treated with 1 mM rotenone as a positive control and 100 µM MitoTempo as a negative control. (**B**) Effect of Gα13 loss on long-term colony formation in non-glycolytic metabolites media in PC3 cells. (**C**) Western blot of stable expression of SOD2 and catalytically inactive SOD2 (Q143A) in monolayer PC3 cells. (**D**,**E**) Effect of SOD2 or SOD2 (Q143A) expression on (**D**) colony formation and (**E**) tertiary spheroid colony formation in PC3 cells after silencing Gα13. (**F**) Western blot of stable knockdown of SOD2 in monolayer LNCaP cells. (**G**–**I**) Effect of SOD2 knockdown on (**G**) colony formation, (**H**) spheroid replating and (**I**) tertiary spheroid colony formation after overexpression of Gα13 in LNCaP cells. All data points represent independent experiments. (**A**–**G**) *p* values calculated by matched, one-way ANOVA with Tukey’s post hoc test. (**H**,**I**) *p* values calculated by paired, two-tailed *t*-tests.

We then tested the hypothesis that Gα13 loss leads to phenotypic deficiency in superoxide scavenging in the mitochondria of prostate cancer cells. To this end, we measured mitochondrial superoxide levels by MitoSOX staining and quantified it by flow cytometry. Under glucose culture in ULA conditions, silencing *GNA13* did not affect mitochondrial superoxide levels [Figure 3A]. We then replaced glucose with a non-glycolytic metabolite galactose to mimic conditions associated with an increase in aerobic respiration and oxidative metabolic stress on the electron transport chain (ETC), which in turn increases superoxide production. Under galactose culture in ULA conditions, we observed an increase in mitochondrial superoxide levels that correlated with the extent of *GNA13* knockdown that was statistically significant in the shGNA13-2 PC3 cells. Unfortunately, the re-expression of Gα13 in PC3 cells slightly but insignificantly rescued mitochondrial superoxide levels [Figure 3A]. 

In addition to SOD2 expressed in the mitochondrial matrix, SOD1 is also expressed in the mitochondrial intermembrane space. Therefore, we investigated whether the increase in mitochondrial superoxide levels induced by loss of Gα13 might be due to changes in SOD1 levels. We found that SOD1 protein levels had little to no change upon modulating Gα13 expression [Supplementary Figure S4]. We then measured superoxide levels in the cytosol where SOD1 is mainly expressed. We found that modulation of Gα13 expression in PC3 cells did not affect cytosolic superoxide levels regardless of glucose or galactose culture conditions [Supplementary Figure S7]. These results indicate a specific effect of Gα13-mediated SOD2 on mitochondrial superoxide levels under oxidative metabolic stress conditions.

SOD2 overexpression has been shown to induce metabolic reprogramming in cancer cells [46,47]. In the mitochondria, superoxide is generated by Complexes I, II and III in the electron transport chain under metabolic stress conditions. All three complexes release superoxide into the matrix where SOD2 is expressed, while only Complex III releases superoxide into the intermembrane space where SOD1 is expressed. Hence, we focused on inducing metabolic stress on Complexes I and II. Glutamate and malate transfer electrons via nicotinamide adenine dinucleotide (NADH) to Complex I in the ETC, and succinate transfers electrons via flavin adenine dinucleotide (FADH2) to Complex II [48]. Therefore, we evaluated the effect of long-term oxidative metabolic stress on soft agar colony formation by replacing 11 mM glucose with non-glycolytic metabolites, namely 10 mM galactose, 10 mM glutamate plus 2 mM malate, and 10 mM succinate. In PC3 cells, silencing of *GNA13* resulted in fewer colonies formed when grown in these non-glycolytic metabolites, while no observable change was detected in sh-control cells [Figure 3B]. These data support a mechanism by which loss of Gα13 in PC3 cells increases sensitivity to oxidative metabolic stress that is induced by non-glycolytic metabolites.

### 2.4. SOD2 Rescues Anchorage Independence Lost upon GNA13 Silencing

To investigate whether SOD2 mediates Gα13-induced cell growth in soft agar, we stably expressed SOD2 in PC3 sh*GNA13*-2 cells [Figure 3C]. Overexpression of SOD2 rescued the suppression of colony and spheroid formation caused by *GNA13* silencing in PC3 cells [Figure 3D,E]. Importantly, colony formation was not rescued when a catalytically-inactive SOD2 (Q143A) mutant was expressed [Figure 3D] [49,50,51,52,53]. Likewise, silencing of *SOD2* expression in LNCaP cells suppressed soft agar colony formation and spheroid formation induced by overexpression of Gα13 in these cells [Figure 3F–I]. Of note, knockdown of *SOD2* in LNCaP cells resulted in cell death and the surviving cells had slow cell proliferation. Hence, there was limited cell lysate for Western blot analysis in Figure 3F. Fortunately, the weak but quantifiable chemiluminescence signal could still be detected and was visible upon higher exposure.

### 2.5. Gα13 Promotes Prostate Cancer Tumor Growth In Vivo

**Figure 4:**
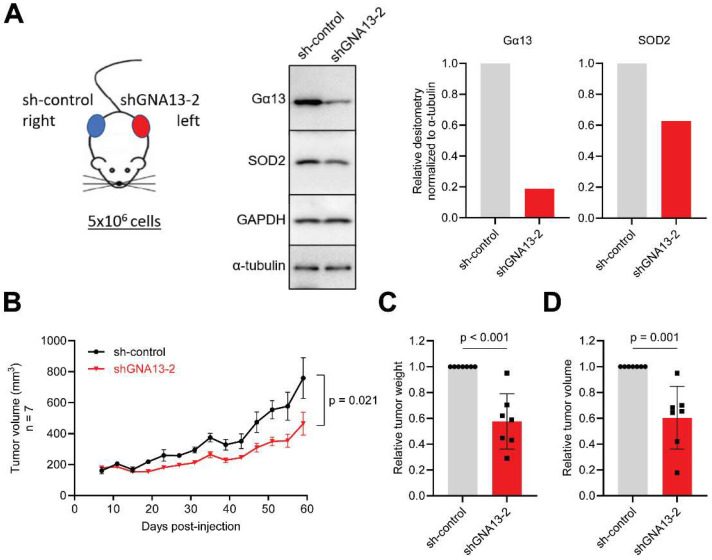
**Loss of Gα13 suppresses PC3 cell tumor growth in NOD SCID mouse xenograft model.** (**A**) Panel 1, schematic of mouse xenograft injection. Panel 2–4, Western blot and relative densitometry of PC3 cells on the day of xenograft injections. (**B**) Tumor growth rate (n = 7). (**C**) Relative tumor weight per mouse. (**D**) Relative tumor volume per mouse. *p* values calculated by unpaired, two-tailed *t*-tests.

To explore the relevance of our in vitro findings on tumor formation in an in vivo model, we generated xenograft tumors using PC3 cells in NOD-SCID mice (see Methods). To examine the tumorigenic effect associated with silencing of *GNA13* in the same animal, we subcutaneously injected 5 × 10^6^ PC3 sh-control or sh*GNA13*-2 cells into the right and left flank, respectively, in each mouse [Figure 4A, left]. At the time of xenograft implantation, SOD2 protein levels were lower in PC3 sh*GNA13*-2 cells by approximately 40% compared to sh-control cells [Figure 4A, right]. Notably, silencing *GNA13* in PC3 cells significantly reduced tumor growth rate and tumor weight by approximately 40% [Figure 4B–D, Supplementary Figure S8A]. Furthermore, we found no significant change in the weight of the mice, and the PC3 sh*GNA13*-2 tumors showed sustained silencing of *GNA13* [Supplementary Figure S8B,C]. In keeping with our in vitro results, these data support a role for Gα13 in tumor formation in vivo.

## 3. Discussion

Several lines of evidence indicate that dominant-active Gα13 promotes cell transformation and can be oncogenic in solid cancers [1,3,54,55]. However, few studies have assessed the effect of the naturally existing wild-type Gα13 on tumorigenic phenotypes in vitro or in vivo [7,12,13]. Here, we showed that Gα13 plays an oncogenic role in prostate cancer cell lines in anchorage-independent in vitro and in vivo models. In addition, we showed that Gα13 regulates SOD2 expression that in turn modulates anchorage-independent growth. Moreover, we showed that Gα13 governs the response to oxidative metabolic stress induced by non-glycolytic metabolites, indicating an important connection between Gα13 and SOD2 in cancer cell phenotypes.

There is limited evidence on the role of Gα13 in mitochondrial processes. Although Gα13 is not present in the mitochondria [56], it has been reported to alter the expression of genes involved in mitochondrial biogenesis, oxidative phosphorylation and insulin insensitivity in normal tissues [57,58,59]. However, the role of Gα13 in the mitochondria in cancer cells and its effect on tumorigenesis is hitherto unrecognized. Our findings that Gα13-regulated genes are enriched in the mitochondria, and that loss of Gα13 increases mitochondrial superoxide levels and suppresses soft agar growth under metabolic oxidative stress conditions, have identified a novel function of Gα13 in the regulation of reactive oxygen species, particularly in response to oxidative metabolic stress in prostate cancer cell lines. Hence, this study highlights a new biological process in which Gα13 regulates mitochondrial superoxide scavenging and reduces oxidative metabolic stress in tumorigenesis.

While no studies have demonstrated a direct signaling pathway or transcription factor between Gα13/Gα12 and antioxidant gene expression, there are some limited studies on Gα13 and antioxidant response element (ARE) activity and the NRF2 transcription factor. One study showed that in mouse embryonic fibroblasts, the Gα13–Rho–PKCδ axis mediated NRF2-activating phosphorylation at serine-40 [22]. In another study, expression of constitutively active Gα13 increased 3×ARE reporter activity in a RhoA-dependent manner and increased NRF2 translocation to the cell nucleus in mouse fibroblast NIH3T3 cells [23]. Even though these studies did not show whether Gα13-mediated NRF2 activation leads to *SOD2* (or other antioxidant gene) expression, other studies showed that NRF2 acts on the *SOD2* promoter through the use of luciferase assays [24] and by ChIP-qPCR analysis using an anti-NRF2 antibody [24], and that NRF2 activation increases *SOD2* mRNA and protein expression [24,25]. Hence, NRF2 is potentially the mediating transcription factor between Gα13 and the *SOD2* promoter and should be explored in future studies.

To study the effect of Gα13-mediated SOD2 expression in prostate cancer biology, we employed methodologies that are different from previous studies on this topic. Both PC3 and LNCaP harbor the *SOD2* (rs4880) polymorphism [40], which we also observed in our transcriptome datasets. Hence, we rescued SOD2 expression by using the cDNA of *SOD2* (rs4880) and also tried to rescue SOD2 protein expression closer to endogenous levels to reflect a more natural state of redox regulation. This is different from previous studies where SOD2 protein levels were increased many-fold above endogenous levels [60,61,62]. Consistent with CRISPR knockout of *SOD2* [63], our study showed that knockdown of *SOD2* results in inhibition of clonogenic cell growth. Furthermore, in contrast to cell growth in monolayer [61], we assessed the effect of SOD2 in anchorage-independent growth models, which better mimic the pathophysiological tumor condition [64,65,66]. In addition, we evaluated both androgen-responsive LNCaP and androgen-independent PC3 cells [60,62,67]; notably, the role of *SOD2* (rs4880) in PC3 clonogenic growth had not been evaluated before [34,60]. In addition to *SOD2* mRNA levels [68], we also investigated *SOD2* promoter activity as well as its mitochondrial protein levels. Importantly, the ability of SOD2 to support anchorage-independent cell survival requires its dismutase activity, as we showed that the catalytically-inactive Q143A mutant of *SOD2* [49,50,51,52,53] cannot rescue the effect of Gα13 loss.

Our study has some limitations. First, we measured superoxide levels by conventional flow cytometry (according to the manufacturer’s protocol); however, recent studies recommend that the superoxide probes dihydroethidium (DHE) and its derivative MitoSOX be quantified by liquid chromatography–mass spectrometry to improve reliability [69]. In our study, we overcame some of the limitations of the MitoSOX stain by using a low concentration (0.5 µM) and by normalizing mitochondrial superoxide levels to the mitochondrial mass, assessed by mitochondrial membrane potential-independent MitoView staining (see Methods). Second, we used a *SOD2* promoter region from −2500 bp to +500 bp from the transcription start site, and our sequence is slightly shorter than those used in other studies, which span from −3340 bp to +260 bp [70] and can be up to +2000 bp [71,72]. This might have affected the sensitivity of our promoter activity in the luciferase assays as additional NFκB and AP-1 binding sites further upstream and the intronic NFκB binding site are missed in our promoter constructs. Despite this, our study detected a suppression of *SOD2* promoter activity upon silencing of *GNA13* in PC3 cells. Lastly, although colony formation in non-glycolytic metabolites was further suppressed in PC3 cells with stable knockdown of *GNA13*, it is unclear whether this is due to increased superoxide levels induced by SOD2 loss or due to defects in mitochondrial respiration induced by Gα13 loss [Supplementary Figure S2]. Regardless, silencing of *GNA13* in PC3 cells decreased cell survival, which was further decreased under oxidative metabolic stress conditions.

## 4. Materials and Methods

### 4.1. Cells and Cell Culture

PC3 (CRL-1435, ATCC) and LNCaP (CRL-1740, ATCC) were cultured in Roswell Park Memorial Institute (RPMI) 1640 medium (Cat. 22400089, Gibco, Grand Island, NY, USA) supplemented with 10% heat-inactivated FBS (Cat. 10500064, Invitrogen, Carlsbad, CA, USA), 1% penicillin/streptomycin (Cat. 15140122, Gibco, Waltham, MA, USA). Cells were maintained at 37 °C with 5% CO_2_ and passaged to a maximum of 10–15 passages for experiments.

Non-glycolytic media were made with glucose-free RPMI 1640 Medium (Cat. 11879020, Gibco, Waltham, MA, USA) supplemented with 25 mM HEPES (Cat. 15630080, Gibco, Waltham, MA, USA), 10% FBS, 1% penicillin/streptomycin and either 10 mM galactose (G5388, Sigma-Aldrich, St. Louis, MO, USA), 10 mM succinic acid (Cat. S9512, Sigma-Aldrich), or 10 mM glutamic acid (Cat. G8415, Sigma–Aldrich, Waltham, MA, USA) plus 2 mM malic acid (Cat. M7397, Sigma–Aldrich, Waltham, MA, USA), and the pH was adjusted to approximately 7.4 with NaOH (Cat. S5881, Sigma, Waltham, MA, USA).

### 4.2. Plasmid Constructs

The cloning method and shRNA sequences against *GNA13* were published previously [15]. PC3 cells with stable expression of sh*GNA13* were maintained in 10 μg/mL blasticidin (Cat. 3513039, Sigma, Waltham, MA, USA). Gα13 rescue expression was performed using *GNA13* wild-type cDNA in the pLVX-puromycin vector. Overexpression of Gα13 in LNCaP cells was performed using *GNA13* wild-type cDNA in the pBabe-puromycin vector. Transduced cells were maintained in 2–5 μg/mL puromycin (Cat. P8833, Sigma, Waltham, MA, USA).

Both PC3 and LNCaP harbor the *SOD2* (rs4880) polymorphism (alanine at position 16) [40]. The *SOD2* (valine at position 16) cDNA was cloned from pBI-EGFP-MnSOD (#16612, Addgene, Watertown, MA, USA) at the XhoI and NheI restriction sites into the pLVX-puromycin vector, which was cut with XhoI and XbaI. The resulting construct was then subjected to site-directed mutagenesis by In-Fusion^®^ Snap Assembly Master Mix (Cat. 638952, Takara, Japan) to replace valine-16 with alanine to create pLVX-SOD2 (alanine at position 16), which is simply referred to as SOD2 in this paper. The pLVX-SOD2 plasmid was further subjected to site-directed mutagenesis to create the catalytically-inactive *SOD2 (Q143A)* mutant [49,50,51,52,53]. The primers used for site-directed mutagenesis are shown in Supplementary Table S1. Overexpression of SOD2 in PC3 cells was performed using pLVX-SOD2 and pLVX-SOD2 (Q143A), and transduced cells were maintained in 2 μg/mL puromycin.

A verified shRNA sequence against *SOD2* was obtained from the pLKO.1-sh*SOD2*-puromycin plasmid (#102976, Addgene) by cutting at the KflI and EcoRI restriction sites and ligated into the pLKO.1-hygromycin vector at the same restriction sites. Stable knockdown of *SOD2* in LNCaP cells was performed using pLKO.1-sh*SOD2*, and transduced cells were maintained in 50 μg/mL hygromycin B (Cat. 09287-84, Nacalai Tesque, Kyoto, Japan).

### 4.3. Soft Agar Colony Formation

PC3 (2500 or 5000 cells/well) or LNCaP (20,000 cells/well) cells were suspended in 0.3% agar and seeded on top of 0.6% agar with complete RPMI medium in 24-well ultra-low attachment (ULA) plates (#3473, Corning, Corning, NY, USA). After 21 days, colonies were stained with 5 mg/mL methylthiazolyldiphenyl tetrazolium bromide (MTT, #M2128, Sigma, St. Louis, MO, USA). Colonies (>40 µm) were counted using GelCount™ (Oxford Optronix Ltd., Abingdon, UK) using a 24-well plate mask at 2400 DPI resolution and CHARM settings appropriate for the cell line.

### 4.4. Spheroid Formation and Serial Re-Plating

PC3 spheroids (1000 cells/well) were generated in different concentrations of growth factor reduced Matrigel^®^ (Cat. 354230, Corning) diluted in complete RPMI in 96-well ultra-low attachment plates (#7007, Corning) and a final concentration of 125 μg/mL was determined as suitable to support spheroid formation [Supplementary Figure S1D] [35,36]. For spheroid serial re-plating experiments, spheroids were generated in 96-well ULA plates (#3474, Corning). Primary, secondary, and tertiary spheroids were trypsinized (0.25% Trypsin-EDTA, Cat. 25200056, Gibco) into single cells and re-plated at a 1:2 ratio in 125 μg/mL Matrigel. Tertiary spheroids were counted by GelCount, then seeded in soft agar for colony formation over 21 days.

### 4.5. Western Blot Analysis

For whole-cell lysates, protein samples were lysed in Tris Lysis Buffer, quantified, and prepared as previously described [73]. Proteins were resolved on SDS–PAGE and transferred to polyvinylidene fluoride (PVDF) membranes (Cat. 1620177, Bio-Rad, Hercules, CA, USA). Primary antibodies were incubated with the membranes overnight at 4 °C followed by secondary antibody incubation and visualization by chemiluminescence with Pierce^®^ ECL (Cat. 32106), or SuperSignalTM West Femto (Cat. 34096, Thermo Fisher Scientific, Waltham, MA, USA). Primary antibodies: anti-Gα13 (6F6-B5, Cat. ST1629, Calbiochem, San Diego, CA, USA), anti-SOD2 (D3X8F, #13141, CST), anti-SOD1 (A01005-40, Genscript), anti-α-tubulin (T5168, Sigma, Waltham, MA, USA), anti-GAPDH (Ab8245, Abcam, Cambridge, UK), anti-VDAC (D73D12, #4661, CST, Waltham, MA, USA), anti-SDHA (D6J9M, #5839, CST, Waltham, MA, USA), and anti-TOMM20 (FL-145, sc11415, Santa Cruz, CA, USA). Secondary antibodies: goat anti-mouse (AP200P, Millipore, San Diego, CA, USA) and goat anti-rabbit (AP132P, Millipore, CA, USA). Densitometry values were obtained using the Volume Tools function in the Image Lab software (Bio-Rad, version 6.0.1).

### 4.6. RNA Sequencing and Analysis

Described in the Supplementary Methods.

### 4.7. Quantitative Real-Time PCR

Total RNA extraction and reverse transcription was performed as previously described [73]. Quantitative real-time PCR of cDNA (25 ng) was performed using iQ™ SYBR^®^ Green Supermix (Cat. 1708880, Bio-Rad, Hercules, CA, USA) on a CFX96 Real-time PCR system (Bio-Rad Laboratories, Hercules, CA, USA) according to the manufacturer’s protocol. Reactions were conducted in triplicate with β-actin (ACTB) as the housekeeping control. The primer sequences are provided in Supplementary Table S1. Relative mRNA expression was determined using the standard 2^−ΔΔCt^ method [74].

### 4.8. Promoter Activity Luciferase Reporter

The *SOD2* promoter region (hg38, ENST00000538183.7) from −2500 bp upstream to +500 bp downstream of the transcription start site was commercially cloned (by Genscript) into the pGL3-basic vector (Promega, Madison, WI, USA) at the KpnI and XhoI restriction sites upstream of the promoter-less firefly luciferase gene.

The methods for luciferase reporter assays have been previously described [7,73]. For basal SOD2 promoter activity, 10^5^ cells were seeded into 6-well plates and co-transfected with 1 ng pRL-TK (HSV-thymidine kinase promoter renilla luciferase control) and 2 μg of either pGL3-*SOD2*-promoter or pGL3-basic vector using jetPRIME transfection reagent (Cat. 71260, Polyplus, Illkirch-Graffenstaden, France) according to the manufacturer’s protocol. Then, at 16 h post-transfection, cells were trypsinized and transferred to 24-well ULA plates. Firefly and renilla luciferase activities were measured 2–4 h later using the Dual Luciferase Assay System (Cat. E1960, Promega) on an Infinite M200 Pro Microplate Reader (Tecan, Switzerland). The ratio of Firefly luciferase activity to Renilla luciferase activity was calculated and the fold change relative to control cells was plotted.

### 4.9. Crude Mitochondria Extraction

Mitochondria were extracted as previously described [75]. Briefly, the cells were resuspended in Buffer A (83 mM sucrose, 10 mM HEPES pH 7.4) with protease inhibitors and 1 mM PMSF and incubated for 10 min on ice. Then, the cells were passed through a 27-gauge needle (Cat. AN2716R1, Terumo, Japan) 20 times. The homogenates were centrifuged at 1000× *g* for 10 min at 4 °C. The supernatant was transferred to a new tube and the remaining pellet was resuspended in Buffer B (250 mM sucrose, 10 mM HEPES, pH 7.4) with protease inhibitors and 1 mM PMSF. The suspension was passed through a 27-gauge needle 20 times and centrifuged again. The supernatants of Buffer A and Buffer B were combined and centrifuged at 12,000× *g* for 15 min at 4 °C. The mitochondrial pellet was resuspended in Buffer C (320 mM sucrose, 1 mM EDTA, 10 mM Tris-HCl, pH 7.4) supplemented with protease inhibitors and 1 mM PMSF.

### 4.10. Mitochondrial Superoxide to Mitochondrial Mass Ratio by Flow Cytometry

First, 1 × 10^6^ cells per well were seeded in 6-well ULA plates and cultured in either 11 mM glucose or 10 mM galactose media for 48 h. Then, 1 × 10^5^ cells per well were seeded into 96-well ULA plates and sequentially stained with 0.5 µM MitoSOX Red (Cat. M36008, Invitrogen), 100 nM MitoView Green (Cat. 70054, Biotium, Fremont, CA, USA) and 1:200 LIVE/DEAD™ Violet (Cat. L34955, Invitrogen) according to the manufacturer’s protocols. Cells were incubated at 37 °C for 15 min with each stain followed by washing twice with 1 × PBS between stains. The samples were analyzed on a BD LSR Fortessa cytometer (BD Biosciences, Franklin Lakes, NJ, USA) within 4 h. The data were acquired on BD FACSDiva software (version 8, Becton Dickinson, USA) and analyzed on Flowjo (version 10.8.1, Becton Dickinson, USA). The relative mitochondrial superoxide to mitochondrial mass ratio was calculated by the median fluorescence intensity (MFI) of MitoSOX Red divided by the MFI of MitoView Green. PC3 sh-control cells were treated with 1 mM rotenone (Cat. R8875, Sigma) as a positive control and 100 µM MitoTempo (Cat. SML0737, Sigma) as a negative control.

### 4.11. Mouse Xenograft

All animal procedures were approved and conducted in accordance with the guiding ethical principles of the Institutional Animal Care And Use Committee at SingHealth, Singapore (IACUC 2019/SHS/1462). Male NOD-SCID mice (7–8 weeks old) were obtained from InVivos (Singapore). The required number of mice was calculated by power analysis using the calculated effect size based on a pilot study (n = 2), power 0.90, and α = 0.05. Mice were housed in single sterile animal cages under laminar flow hoods in a temperature-controlled room with a 12 h light/dark schedule and fed autoclaved chow and water ad libitum.

The PC3 cells stably expressing the sh-control or sh*GNA13*-2 were prepared with 5 × 10^6^ cells in 100 μL Matrigel and were injected subcutaneously into the right and left flank, respectively. Tumor volumes were measured every 4 days for 60 days starting on day 7 post-injection. The longest and two shortest diameters of the tumors were measured with electronic vernier calipers (Cat. 14-648-17, Thermo Fisher Scientific) and tumor volume (cm^3^) was calculated using the following formula: (the sum of the two shortest diameters)/2 × (the longest diameter) × π/6. Mice were euthanized with CO_2_ followed by cervical dislocation. The tumors were excised, snap-frozen in liquid nitrogen and stored at −80 °C.

### 4.12. Statistical Analysis

All in vitro experiments were repeated independently at least three times. Values are expressed as means ± standard error of means (SEMs). *p*-values were calculated using *t*-tests (two-tailed) or one-way analysis of variance (ANOVA) with post hoc correction for multiple comparisons and using the pooled mean values across 2–10 technical replicates within each independent experiment. For the mouse experiments, *p*-values are calculated by paired *t*-tests (two-tailed). Statistical analyses were performed using GraphPad Prism 10. A *p*-value < 0.05 was considered statistically significant.

## 5. Conclusions

In summary, we propose a novel biological route of Gα13-mediated anchorage-independent growth and response to oxidative metabolic stress through regulation of SOD2 expression in prostate cancer cells. Given that SOD2 protein levels correlate with prostate cancer Gleason grade, identifying the upregulated GPCRs that signal through Gα13 in prostate cancer could lead to novel preventive or therapeutic strategies. More detailed studies are needed to define the underlying mechanisms associated with the Gα13-regulated response to oxidative metabolic stress in prostate cancer. 

This article is a revised and expanded version of a Conference Abstract entitled Gα13 Promotes Clonogenic Growth By Increasing Tolerance To Oxidative Metabolic Stress In Prostate Cancer Cells, which was presented at the 2024 Endocrine Society Annual Meeting, Boston, MA, USA, Presentation 1 June 2024 [76].

## Data Availability

The data that support the findings of this study are available from the corresponding author upon a reasonable request. All RNA-seq data are openly available in GEO datasets at https://www.ncbi.nlm.nih.gov/gds (GSE133893, GSE263633).

## Supplementary Materials

The following supporting information can be downloaded at: https://www.mdpi.com/article/10.3390/ijms26104883/s1. References [77,78,79,80,81,82,83,84,85,86,87,88,89,90,91,92,93] are cited in the Supplementary Materials.

## Abbreviations

The following abbreviations are used in this manuscript:

ANOVA	one-way analysis of variance
ARE	antioxidant response element
CRPC	castration-resistant prostate cancer
DHE	dihydroethidium
ETC	electron transport chain
FADH2	flavin adenine dinucleotide
GNA13	guanine nucleotide-binding protein subunit alpha-13
GPCR	G protein-coupled receptor
IACUC	Institutional Animal Care and Use Committee
MFI	median fluorescence intensity
MTT	methylthiazolyldiphenyl tetrazolium bromide
NADH	nicotinamide adenine dinucleotide
NRF2	nuclear factor erythroid 2-related factor 2
PCR	polymerase chain reaction
PVDF	polyvinylidene fluoride
RPMI	Roswell Park Memorial Institute
SDS-PAGE	sodium dodecyl sulfate-polyacrylamide gel electrophoresis
SEM	standard error of mean
SOD2	superoxide dismutase 2
SU2C/PCF	Stand Up to Cancer/Prostate Cancer Foundation
TCGA	The Cancer Genome Atlas
ULA	ultra-low adherent
