# Supplementary material for: Gα13 Promotes Clonogenic Growth by Increasing Tolerance to Oxidative Metabolic Stress in Prostate Cancer Cells
Source: Int J Mol Sci. 2025 May 20;26(10):4883. doi: 10.3390/ijms26104883 (PMC12111955; doi:10.3390/ijms26104883)
Supplement: Supplementary file 1 [file ijms-26-04883-s001.zip › ijms-3547434-supplementary.pdf]

International Journal of *Molecular Sciences*

MDPI

Supplementary Information

Gα13 promotes clonogenic growth by increasing tolerance to oxidative metabolic stress in prostate cancer cells.

Di Wu, Wei Kiang Lim, Xiaoran Chai, Veerabrahma Pratap Seshachalam, Suhail Ahmed Kabeer Rasheed, Sujoy Ghosh and Patrick J. Casey

1. Supplementary Experimental Methods

1.1. RNA sequencing

The methods for RNA sequencing in PC3 cell lines were previously described in detail (GEO DataSets GSE133893)[1]. The samples include PC3 sh-control, shGNA13-1 and shGNA13-2, and the transcript read counts are expressed as Fragments Per Kilobase of transcript per Million mapped reads (FPKM). PC3 shGNA13-1 versus sh-control, and shGNA13-2 versus sh-control cells gene expression fold changes were calculated using Cufflinks version 2.2.1[2,3], as previously reported[1].

RNA sequencing was performed in LNCaP cell lines stably expressing pBabe-vector and pBabe-Gα13. LNCaP cells (3×10^6) were seeded in 10-cm plates in 3 biological replicates and cultured for 48 h until the cells reached 80% confluency. Total RNA was extracted (RNeasy mini kit, Qiagen) for paired-end sequencing of 150-nucleotide read length on the Novaseq 6000 next generation sequencing platform (Novogene, Singapore). Sequencing read quality was assessed via FastQC software (version 0.11.7)[4]. Reads were mapped to the human reference transcriptome (Gencode version 38), via the STAR aligner[5] and reads were aggregated into gene counts via the featureCounts function in the Rsubread R package 9 (version 1.30.9)[6]. Differences in library sizes among the samples were adjusted via TMM normalization[7] and potential sample outliers were evaluated via Principal Component Analysis (PCA) through the princomp function in R. Differential gene expression analysis was conducted via the limma software [8]. The transcript read counts are expressed as counts per million (CPM) in the $\log_2$ scale.

Note that we used Cufflinks-based gene quantitation and fold-change/statistical significance estimation for the PC3 cells as only single samples were available in each group, and Cufflinks is suitable for such single sample comparisons. In contrast, we had replicate samples available for the LNCaP cells and therefore we utilized the more powerful count based approaches available through limma. However, we focused all downstream analysis only on the genes and pathways that were identified in common in both cell types, to remove any analysis- pipeline specific effects.

1.2. DAVID over-representation analysis

To gain insight on the biological processes that regulate Gα13-mediated phenotypes in prostate cancer cells, we performed RNA sequencing of the PC3 (sh-control, shGNA13-1, and shGNA13-2) and LNCaP cells (pBabe-vector and Gα13). We performed two types of pathway enrichment analysis (over-representation analysis and gene-set enrichment analysis) to increase confidence in our results. First, we performed over-representation analysis to identify the common Gα13-mediated processes between PC3 and LNCaP cells. In PC3 cells, the change in expression of 9420 genes were concurrent upon Gα13 knockdown by both shRNAs (shGNA13-1 and shGNA13-2), but only 3077 genes displayed fold-changes that were concordant with the degree of Gα13 knockdown by each shRNA. To identify the common Gα13-mediated biological processes, we combined the PC3 and LNCaP datasets and identified 1195 genes that were mutually regulated by Gα13. The 1195 genes were analyzed against the Gene Ontology (GO)[9,10] Cellular Component (GOTERM_CC_ALL) gene sets using DAVID Analysis Wizard Version 2022[11,12].

1.3. Gene Set Enrichment Analysis (GSEA)

Since the PC3 dataset only has single sample per group, we first identified 19,555 genes that were modulated by Gα13 in the same direction of fold change (up- or down-regulation) in the shGNA13-1 and shGNA13-2 compared to sh-control. Then, for each comparison, we generated a combined metric according to the formula: $\log_2$(fold change) × $-\log_{10}$(p value), as previously reported[13]. The PC3 ranked gene list was analyzed using GSEA[14,15] software version 4.3.2 against the GO Biological Processes[9,10] gene sets and the MitoPathways gene sets from MitoCarta[16-18].

LNCaP-vec and LNCaP-Gα13 cells expressed 17,297 genes and the CPM counts were uploaded into GSEA and similarly analyzed against GO Biological Processes.

1.4. Cell proliferation assay in monolayer

1×10^3 PC3 cells were seeded in 96-well solid white plates (#3917, Corning). Cell proliferation was evaluated by luminescent cell viability assay CellTiter-Glo (Cat. G7571, Promega, Madison, WI, USA). At each time point every 24 h, an equal volume (100 µl) of CellTiter-Glo reagent the was added to each well. The plates were placed on an orbital shaker (Cat.PSU-10i, Biosan, Latvia) at 400 rpm for 5 min. The luminescence was measured at 10 mins after adding CellTiter-Glo using a Infinite M200 Pro Microplate Reader (Tecan, Switzerland).

2.5×10^3 PC3 or LNCaP cells were seeded in 96-well clear bottom plates (#3595, Corning). Cell confluency was calculated by IncuCyte ZOOM™ (Essen BioScience) time-lapse live-cell microscopy system.

1.5. Protein extraction from PC3 spheroids

100 µl of 0.25% Trypsin-EDTA (Cat. 25200-056, Gibco, Canada) was added to PC3 spheroids that were cultured in 125µg/ml Matrigel in 96-well ultra-low adherent plates. Spheroids were resuspended and transferred to 1.5 ml tubes and centrifuged at 5000 rpm for 3 min. Media was removed and cell pellets were resuspended in 100 µl of trypsin and centrifuged again. Cell pellets were resuspended and lysed in Tris lysis buffer. Spheroid cell lysates were quantified for protein amount by Pierce™ BCA protein assay kit (Thermo Scientific) and resolved by SDS-PAGE.

1.6. si-GNA13 transfection

Transfection of siRNA was performed using JETPRIME transfection reagent (Cat. 114-07, Polyplus, France) according to the manufacturer's protocol. 2×10^5 PC3 parental cells were seeded in 6-well plates and transfected with 100 nM si-GNA13 (pool of 4, Cat. L-009948-00-0005, Dharmacon, Lafayette, Colorado, USA) and scramble control (D-001810-10-50, Dharmacon). Culture media was replaced 6-16 h post-transfection. Cells were trypsinized and transferred to 24-well ultra-low adherent plates for more than 6 h. Total RNA was extracted and cDNA was synthesized to perform quantitative real-time PCR for GNA13 and SOD2 mRNA expression.

1.7. Cytosolic superoxide staining

1×10^6 cells were seeded in 6-well ultra-low adherent plates. 48 h later, cells were transferred to 96-well ultra-low adherent plates in duplicates and stained with 5µM dihydroethidium (DHE, Cat. D11347, Invitrogen™) in the dark at 37°C for 30 min. Cells were washed with PBS buffer and resuspended in FACS buffer. Samples were analyzed on BD LSR Fortessa cytometer (BD Biosciences) within 4 h. The data was acquired on BD FACSDiva software and analyzed on Flowjo (version 10). DHE fluorescence intensity was obtained at excitation/emission 405/605 nm. Relative cytosolic superoxide was plotted as the median fluorescence intensity (MFI) of DHE relative to PC3 sh-control cells.

Table 1. Primer sequences. All primers were obtained from Integrated DNA Technologies, Singapore.

Human Gene	Forward primer (5′ - 3′)	Reverse primer (5′ - 3′)
	qPCR primers	
GNA13	TCCACCTTCCTGAAGCAGATGC	GCTTCTCTCGAGCATCAACCAG
SOD2 (from Ori-gene)	CTGGACAAACCTCAGCCCTAAC	AACCTGAGCCTTGGACACCAAC
ACTB	TGGCACCCAGCACAATGAA	CTAAGTCATAGTCCGCCTAGAA-GCA
	Site directed mutagenesis cloning primers	
SOD2 (rs4880)	GGCTCCGGCTTTGGGG-TATCTGGGCTCCAGG	CCCAAAGCCG-GAGCCAGCTGCCTGCTGG
SOD2 (rs4880) Q143A	TCCAAATGCGGATCCACTG-CAAGGAACAACAG	GGATCCGCATTTGGACAAGCAG-CAATTTG

2. Supplementary Figures

2.1. Supplementary Figure1

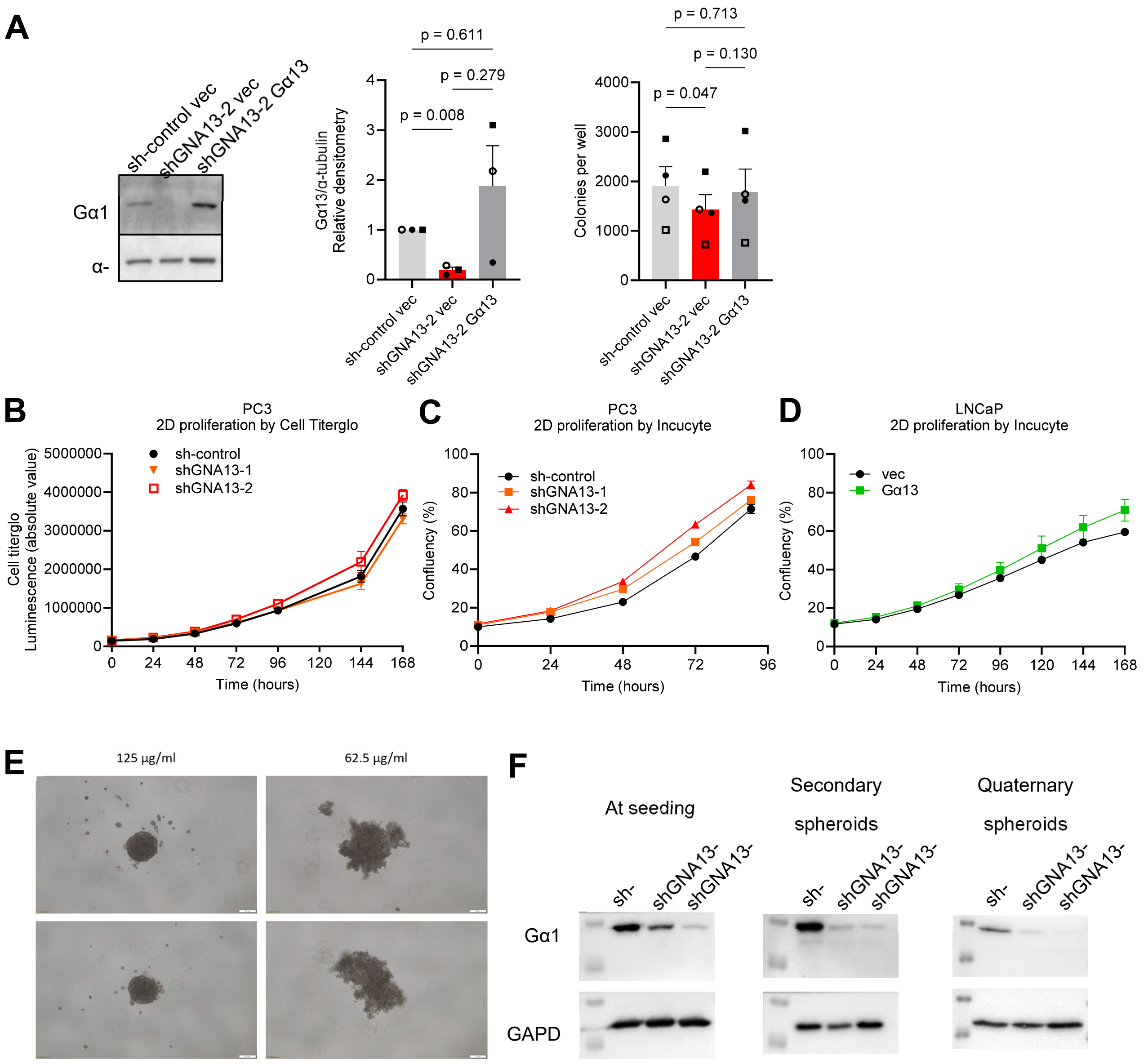

Supplementary Figure S1. Effect of modulating Gα13 on anchorage-independent and monolayer cell growth in PC3 and LNCaP cells.

(A) Gα13 protein levels and the number of colonies formed in soft agar upon rescue expression of Gα13 in PC3 cells. Data points represent independent experiments.

(B) PC3 cells were seeded at 1000 cells per well in 96-well plate and cell viability was measured with Cell Titerglo at 24-h intervals. Experiments were performed with 4-5 technical replicates in three independent experiments. One representative growth curve is shown.

(C) PC3 cells were seeded at 1000 cells per well in 96-well plate and cell confluency was monitored by Incucyte. Experiments were performed with 4 technical replicates in three independent experiments. One representative growth curve is shown.

(D) LNCaP cells were seeded at 2500 cells per well in 96-well plate and cell confluency was monitored by Incucyte. Experiments were performed with 4 technical replicates in three independent experiments. One representative growth curve is shown.

(E) PC3 sh-control cells were seeded at 1000 cells per well in round-bottom ultra-low adherent 96-well plates. Images were taken with an inverted microscope.

(F) Protein lysates were obtained from PC3 spheroids during each re-plating. Gα13 protein levels were quantified by western blot.

2.2. Supplementary Figure2

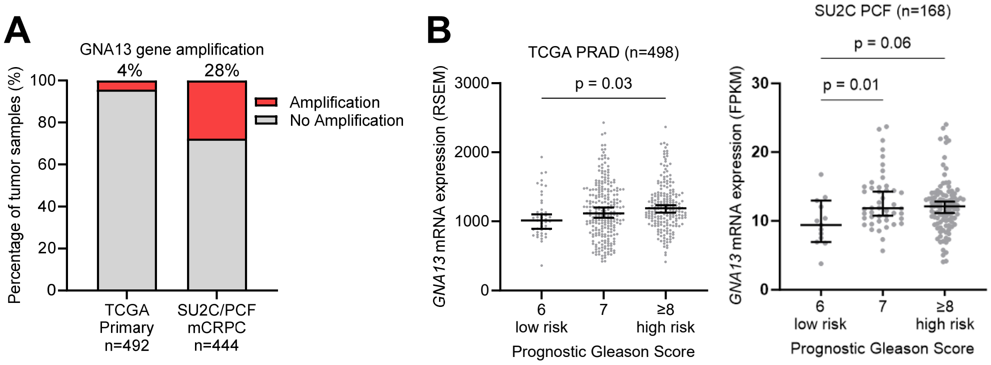

Supplementary Figure S2. ***GNA13*** **mRNA expression in prostate cancer patient samples from exome sequencing data sets.** **(A)** Percentage of *GNA13* gene amplification in primary prostate adenocarcinoma samples from The Cancer Genome Atlas (TCGA; 4% (21/492)) and in metastatic castration-resistant prostate cancer (mCRPC) in the SU2C/PCF (8% (123/444) data set sets. **(B)** Correlation of *GNA13* mRNA levels to prognostic Gleason scores in the TCGA-PRAD and SU2C/PCF.

2.3. Supplementary Figure3

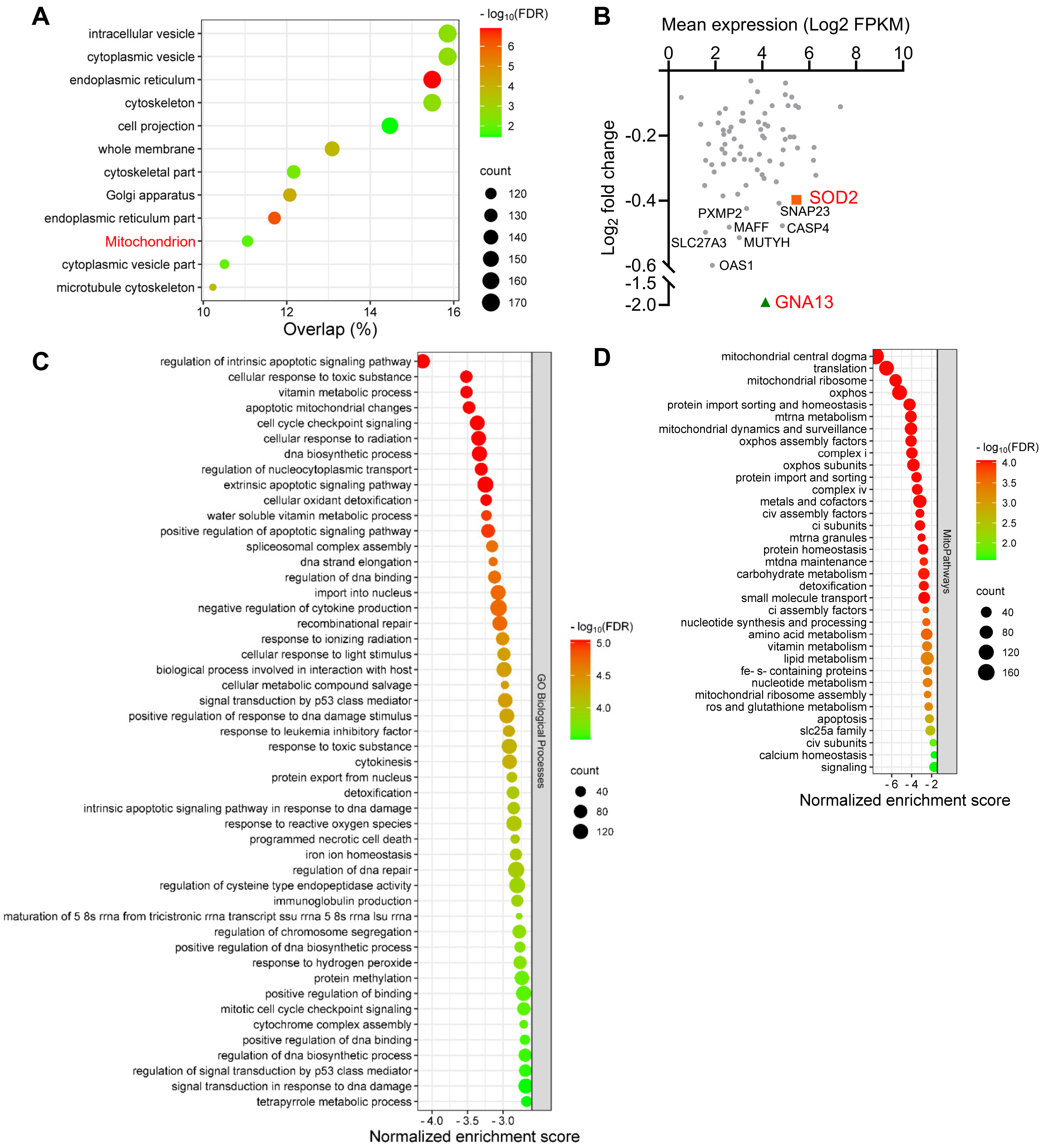

Supplementary Figure S3. Transcriptome analysis of Gα13-mediated gene expression in PC3 and LNCaP cells. .

(A) DAVID over-representation analysis of GO cellular components of the 1195 genes concurrently regulated by Gα13 in PC3 and LNCaP cells (FDR<0.05, gene set size 10-200).

(B) Mean average plot of the genes that are positively regulated by Gα13 in the mitochondria in PC3 cells. As a role for Gα13 in mitochondria processes was unrecognized, we extracted the 120 genes from the mitochondria component and assessed the genes positively regulated by Gα13 in PC3 cells. Among the top ten Gα13-mediated genes that function in the mitochondria, the *SOD2* gene has been reported to correlate with prostate cancer risk[19-22] and prognostic Gleason scores[23,24]. Mean averages are calculated from the shGNA13-1 and shGNA13-2 datasets.

(C) Top 50 common GO biological processes by gene set enrichment analysis in PC3 and LNCaP cells after modulating Gα13 expression. To increase confidence in the pathway over-representation analysis results, we performed gene-set enrichment analysis on the full transcriptome of PC3 and LNCaP separately using GSEA[29,30]. There were 993 GO Biological Processes that are mutually positively regulated by Gα13 in PC3 and LNCaP cells. The top 50 Gα13-mediated biological processes (FDR<0.003) in PC3 cells are shown, which notably includes 'Cellular Oxidant Detoxification', 'Response to Reactive Oxygen Species', 'Reactive Oxygen Species Metabolic Process', and these include SOD2.

(D) Top 30 common MitoPathways (MitoCarta) by gene set enrichment analysis in PC3 cells after knockdown of Gα13. The combined rank of the PC3 transcriptomes (19,556 genes)(shGNA13-1 versus sh-control and shGNA13-2 versus sh-control) was analyzed by GSEA against MitoPathway gene sets from MitoCarta.

2.4. Supplementary Figure4

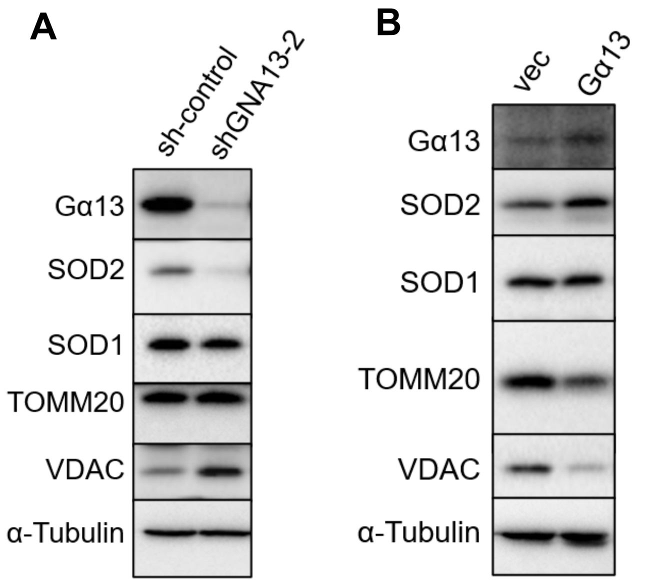

Supplementary Figure S4. SOD proteins and mitochondrial markers in whole cell lysate of PC3 and LNCaP cells.

Western blot of whole cell lysate from (A) PC3 and (B) LNCaP cells showing the change in mitochondrial outer membrane markers TOMM20 and VDAC, and SOD proteins.

2.5. Supplementary Figure5

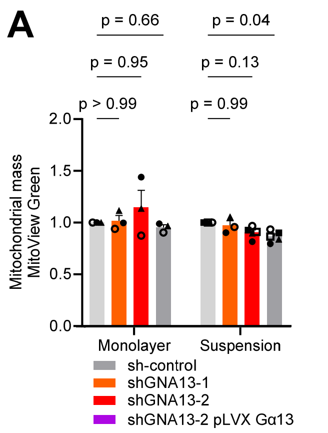

Supplementary Figure S5. The effect of Gα13 on mitochondrial mass.

Table 13. on mitochondrial mass in cells cultured in monolayer and in ultra-low adherent conditions and stained by Mitoview Green (mitochondrial membrane potential independent) in **(A)** PC3 and **(B)** LNCaP cells. Fluorescence readings were obtained by Tecan plate reader. Data is relative to PC3 sh-control and LNCaP pBabe-vector readings under each culture condition. Each data point represents one independent experiment performed with 2-4 technical replicates. P values were determined by matched one-way ANOVA with Tukey test for multiple comparison.

2.6. Supplementary Figure6

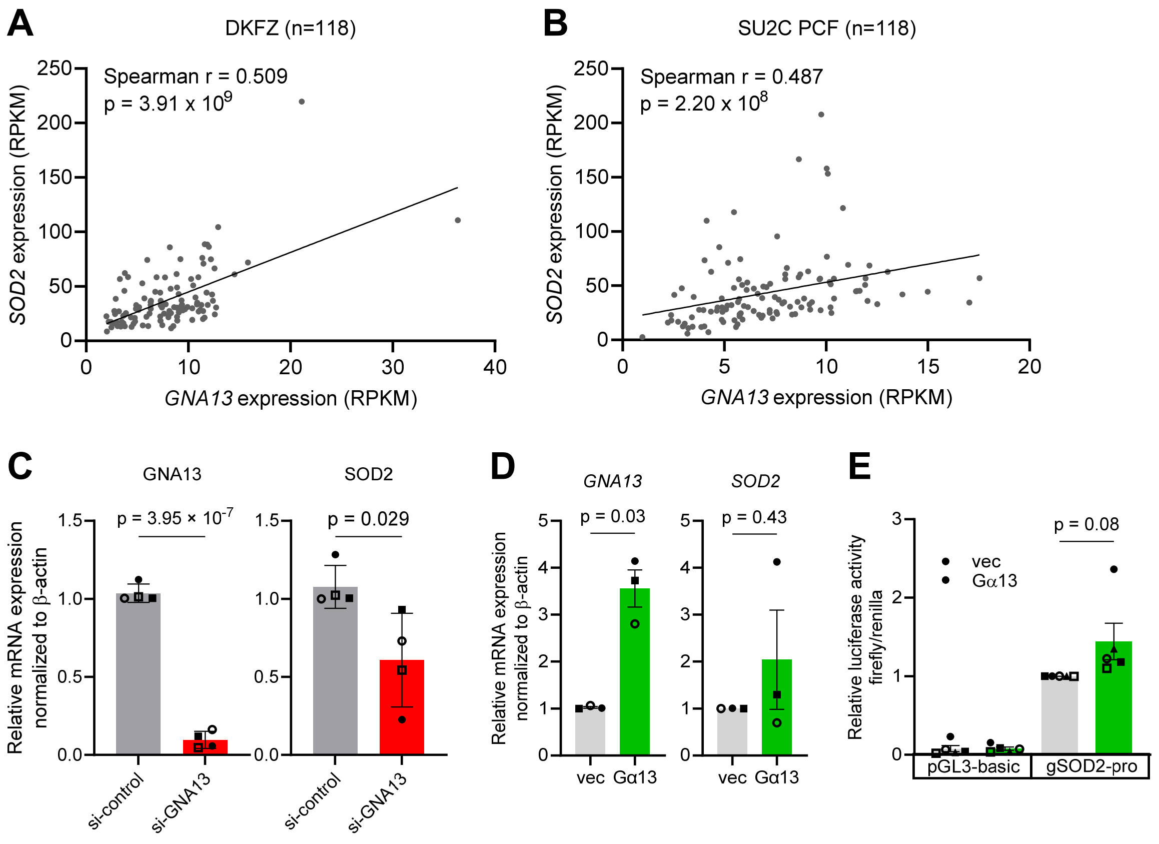

Supplementary Figure S6. Effect of *GNA13* on *SOD2* expression.

(A-B) Prostate cancer patient RNAseq data from (A) the DKFZ study and (B) the SU2C PCF study were extracted from cBioPortal. The mRNA expression of *SOD2* against *GNA13* from prostate

cancer patient tumor samples is plotted. Black line is drawn by linear regression and correlation is calculated by Spearman's Rho.

(C) Effect of transient silencing of *GNA13* on *SOD2* mRNA expression in PC3 parental cells. siRNA against *GNA13* transfected into PC3 parental cells using Lipofectamine. 16 h post-transfection, cells were transferred to ultra-low adherent conditions for 24 h. mRNA expression was normalized to *ACTB* mRNA expression.

(D) Effect of Gα13 on *SOD2* mRNA expression in LNCaP cells.

(E) Effect of Gα13 on *SOD2* promoter activity in LNCaP cells.

(C-E) Data points represent independent experiments.

2.7. Supplementary Figure7

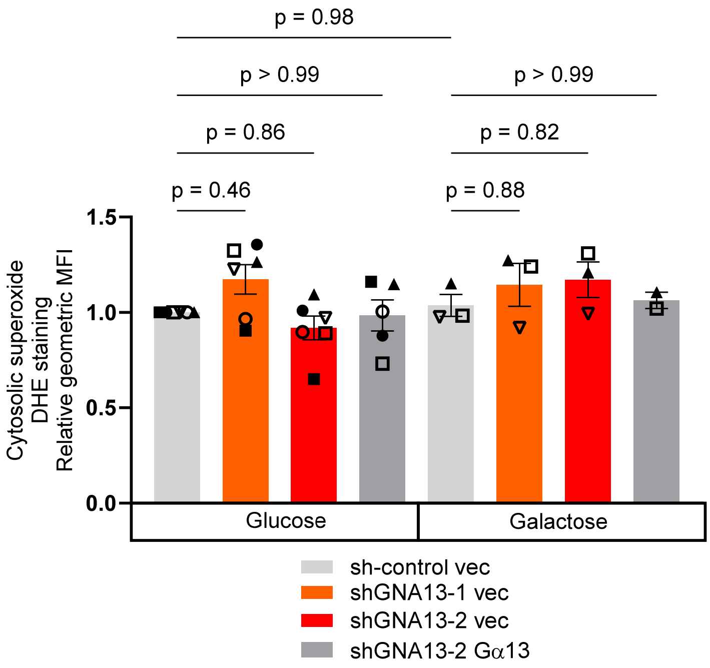

Supplementary Figure S7. Effect of Gα13 on cytosolic superoxide levels in PC3 cells.

PC3 cells were cultured in complete RPMI containing 11mM glucose or 10mM galactose in ultra-low adherent conditions for 48 h. Cells were stained with 5μM dihydroethidium (DHE) in the dark at 37°C for 30 min, per manufacturer's protocol, and fluorescence intensity was acquired by flow cytometry. Data shown is relative to PC3 sh-control cells cultured in normal glucose media. Each datapoint represents an independent experiment. P-values are calculated by one-way ANOVA and multiple comparison are corrected by Tukey's test.

2.8. Supplementary Figure8

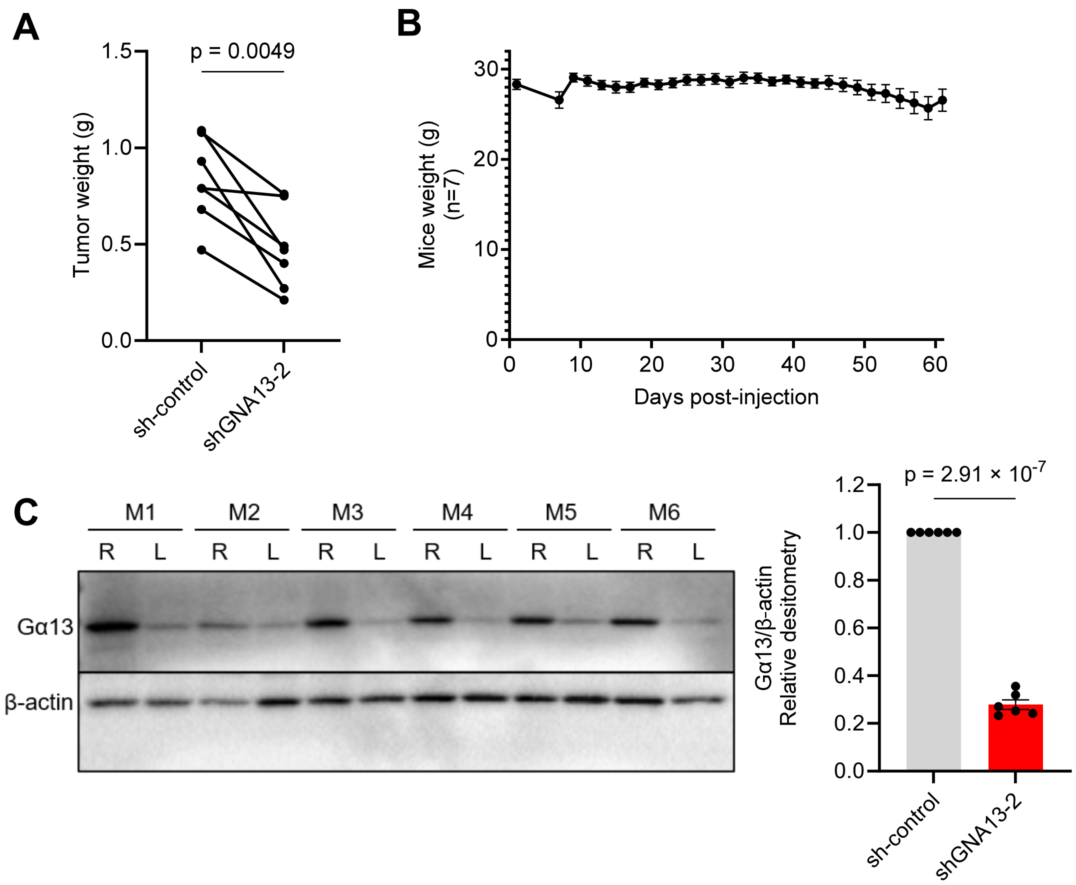

Supplementary Figure S8. Mouse Xenograft.

(A) Tumor weight at 61 days post-injection. Connecting lines indicate the pair of PC3 tumors harvested from the same mouse (n=7 pairs).

(B) Weight of mice over 61 days post-injection of PC3 tumor xenografts.

(C) Western blot of tumor lysates and densitometry plot of Gα13 normalized against β-actin. Mouse#7 died unexpectedly one day prior tumor harvest, hence the necrotic tumors yielded no meaningful data on western blot and was excluded from the final blot.

P-values were calculated by two-tailed, paired T-test.

References

1. Lim WK, Chai X, Ghosh S, et al. Gα-13 induces CXC motif chemokine ligand 5 expression in prostate cancer cells by transactivating NF-κB. *J Biol Chem.* 2019;294(48):18192-18206.
2. Trapnell C, Hendrickson DG, Sauvageau M, Goff L, Rinn JL, Pachter L. Differential analysis of gene regulation at transcript resolution with RNA-seq. *Nat Biotechnol.* 2013;31(1):46-53.
3. Trapnell C, Williams BA, Pertea G, et al. Transcript assembly and quantification by RNA-Seq reveals unannotated transcripts and isoform switching during cell differentiation. *Nat Biotechnol.* 2010;28(5):511-515.
4. Andrews S. FASTQC. A quality control tool for high throughput sequence data. Available online at: http://www.bioinformatics.babraham.ac.uk/projects/fastqc. In:2010.
5. Dobin A, Davis CA, Schlesinger F, et al. STAR: ultrafast universal RNA-seq aligner. *Bioinformatics.* 2013;29(1):15-21.
6. Liao Y, Smyth GK, Shi W. The R package Rsubread is easier, faster, cheaper and better for alignment and quantification of RNA sequencing reads. *Nucleic Acids Res.* 2019;47(8):e47.
7. Robinson MD, Oshlack A. A scaling normalization method for differential expression analysis of RNA-seq data. *Genome Biol.* 2010;11(3):R25.
8. Ritchie ME, Phipson B, Wu D, et al. limma powers differential expression analyses for RNA-sequencing and microarray studies. *Nucleic Acids Res.* 2015;43(7):e47.
9. Ashburner M, Ball CA, Blake JA, et al. Gene ontology: tool for the unification of biology. The Gene Ontology Consortium. *Nat Genet.* 2000;25(1):25-29.
10. Gene Ontology Consortium. The Gene Ontology resource: enriching a GOld mine. *Nucleic Acids Res.* 2021;49(D1):D325-d334.
11. Sherman BT, Hao M, Qiu J, et al. DAVID: a web server for functional enrichment analysis and functional annotation of gene lists (2021 update). *Nucleic Acids Res.* 2022;50(W1):W216-w221.
12. Huang da W, Sherman BT, Lempicki RA. Systematic and integrative analysis of large gene lists using DAVID bioinformatics resources. *Nat Protoc.* 2009;4(1):44-57.
13. Xiao Y, Hsiao TH, Suresh U, et al. A novel significance score for gene selection and ranking. *Bioinformatics.* 2014;30(6):801-807.
14. Mootha VK, Lindgren CM, Eriksson KF, et al. PGC-1alpha-responsive genes involved in oxidative phosphorylation are coordinately downregulated in human diabetes. *Nat Genet.* 2003;34(3):267-273.
15. Subramanian A, Tamayo P, Mootha VK, et al. Gene set enrichment analysis: a knowledge-based approach for interpreting genome-wide expression profiles. *Proc Natl Acad Sci U S A.* 2005;102(43):15545-15550.
16. Calvo SE, Clauser KR, Mootha VK. MitoCarta2.0: an updated inventory of mammalian mitochondrial proteins. *Nucleic Acids Res.* 2016;44(D1):D1251-1257.
17. Pagliarini DJ, Calvo SE, Chang B, et al. A mitochondrial protein compendium elucidates complex I disease biology. *Cell.* 2008;134(1):112-123.
18. Rath S, Sharma R, Gupta R, et al. MitoCarta3.0: an updated mitochondrial proteome now with sub-organelle localization and pathway annotations. *Nucleic Acids Res.* 2021;49(D1):D1541-d1547.
19. Álvarez-González B, Porras-Quesada P, Arenas-Rodríguez V, et al. Genetic variants of antioxidant and xenobiotic metabolizing enzymes and their association with prostate cancer: A meta-analysis and functional in silico analysis. *Sci Total Environ.* 2023;898:165530.
20. Djokic M, Radic T, Santric V, et al. The Association of Polymorphisms in Genes Encoding Antioxidant Enzymes GPX1 (rs1050450), SOD2 (rs4880) and Transcriptional Factor Nrf2 (rs6721961) with the Risk and Development of Prostate Cancer. *Medicina (Kaunas).* 2022;58(10).
21. Zhang LF, Xu K, Tang BW, et al. Association between SOD2 V16A variant and urological cancer risk. *Aging (Albany NY).* 2020;12(1):825-843.
22. Woodson K, Tangrea JA, Lehman TA, et al. Manganese superoxide dismutase (MnSOD) polymorphism, alpha-tocopherol supplementation and prostate cancer risk in the alpha-tocopherol, beta-carotene cancer prevention study (Finland). *Cancer Causes Control.* 2003;14(6):513-518.
23. Miar A, Hevia D, Muñoz-Cimadevilla H, et al. Manganese superoxide dismutase (SOD2/MnSOD)/catalase and SOD2/GPx1 ratios as biomarkers for tumor progression and metastasis in prostate, colon, and lung cancer. *Free Radic Biol Med.* 2015;85:45-55.
24. Quirós I, Sáinz RM, Hevia D, et al. Upregulation of manganese superoxide dismutase (SOD2) is a common pathway for neuroendocrine differentiation in prostate cancer cells. *Int J Cancer.* 2009;125(7):1497-1504.
